# Analysis of transcriptome and differential expression of different types of calli of *Eucalyptus urophylla* *× Eucalyptus grandis*

**DOI:** 10.1371/journal.pone.0322224

**Published:** 2025-05-08

**Authors:** Yamei Liu, Chaohong Wang, Lejun Ouyang, Limei Li, Min Su

**Affiliations:** 1 College of Biological and Food Engineering, Guangdong University of Petrochemical Technology, Maoming, China; 2 College of Life and Geographic Sciences, The Key Laboratory of Ecology and Biological Resources in Yarkand Oasis at Colleges and Universities under the Department of Education of Xinjiang Uygur Autonomous Region, Kashi University, Kashi, China; United Arab Emirates University, UNITED ARAB EMIRATES

## Abstract

*Eucalyptus urophylla X Eucalyptus grandis* is an important afforestation hybrid clone that supports wood safety in China. To explore the mechanism of callus formation and differentiation of different types of *E. urophylla × E. grandis*, we cultivated four different types of *E. urophylla × E. grandis* calli, measured their enzyme activities and endogenous hormones, and sequenced them at the transcriptome level. Transcriptome analysis revealed that there were significant differences in the clustering of differentially expressed genes. Compared with the green calli, 2203, 2485, and 2078 differentially expressed genes were identified in the red, white, and yellow calli, respectively. Differentially expressed genes were involved in metabolic processes, biological regulation, signal transduction, stimulus response, catalytic activity, and binding, such as GRFs gene. Combined with the changes in physiological indices and transcription levels, we revealed the regulatory characteristics of substance storage and antioxidant capacity in the process of callus differentiation of *E. urophylla × E. grandis*, which contributes to the understanding of the mechanism of plant cell growth and differentiation.

## Introduction

The genetic background of *Eucalyptus* is complex, and the relationship between genotype and phenotype is often unclear as most traits are regulated by multiple genes. Conventional breeding methods cannot meet the requirements for cultivating excellent *Eucalyptus* varieties for different purposes [1]. *Eucalyptus* genetic engineering methods can effectively compensate for the shortcomings of sexual reproduction methods, shortening the breeding cycle, and improving the breeding efficiency [2]. Establishing an efficient regeneration and genetic transformation system is a prerequisite for *Eucalyptus* genetic engineering research [3]. Differences in *Eucalyptus* genotypes, explant culturing materials, and the proportions of various hormones are important factors affecting *Eucalyptus* tissue culture regeneration. At present, *E. urophylla × E. grandis* is an important afforestation hybrid clone that exhibit obvious heterosis when planted in a large area. It has a good wood material, strong stress resistance, wide adaptability, fast growth speed, and high yield, and is widely favored by forest operators [4]. The quality of *E. urophylla × E. grandis* calli affects bud differentiation and transformation efficiency. Callus regeneration depends on miRNA regulation [5].

Many miRNAs such as miR156, miR159, miR160, miR164, miR166, miR169, miR171, miR399, and miR482 may participate in *Eucalyptus* somatic embryogenesis and affect embryonic callus development [6–10]. Differentially expressed genes in the callus include those related to auxin, embryogenesis, ethylene, ribosomal protein (RP), zinc finger protein (ZFP), histones, heat shock protein (HSP), and various transcription factors (TFs), such as ARR and GRF [11,12]. However, when and how related genes are expressed in the process of callus formation and development in *Eucalyptus* remains poorly understood.

In this study, we aimed to investigate the transcriptome profiles of different types of *E. urophylla × E. grandis* calli cultured under the same culture medium and conditions. We also aimed to identify the genes involved in the callus development of *E. urophylla* × *E. grandis* and their ability to control the callus reproduction and differentiation. Mining and analyzing transcriptome data from different types of calli of *E. urophylla × E. grandis* is not only beneficial for current *Eucalyptus* breeding programs but also provides valuable resources for future research.

## Materials and methods

### Declaration

We complied with the IUCN Policy Statement on Research Involving Species at Risk of Extinction and the Convention on the Trade in Endangered Species of Wild Fauna and Flora.

### Plant material

Aseptic shoots from *Eucalyptus urophylla X Eucalyptus grandis* clone were provided by Zhanjiang Forestry Science Research Institute.

### Callus induction

The aseptic shoots of *E. urophylla × E. grandis* were cut into 0.5 cm stem segments without bud spots and placed on the autoclaved callus induction medium (Murashige and Skoog medium, 30 g/L sucrose, 7 g/L agar, 1 mg/L PBU, 0.05 mg/L IAA, 100 mg/L VC) at pH 5.8–6.0 for two weeks at 25 °C to induce yellow callus. Some of them continued to be cultured under the same conditions, after one week, the callus differentiated into white. Some calli were transferred to bud differentiation on 1/2 MS medium with 0.3 mg/L 2, 4-D, 2.5 mg/L 6-BA, 100 mg/L VC at pH 5.8–6.0 and cultured in darkness at 25 °C for one week. The calli were differentiated in green. The remaining yellow calli were removed for light culture (light intensity: 2000–2500 LX) and then differentiated into red after one week. The green, red, white, and yellow calli of *E. urophylla × E. grandis* were divided into groups A, B, C, and D, respectively. Three biological replicates of four different types calli were labeled G-, R-, Y-1, 2, or 3 respectively.

### Enzyme extraction and activity assays

Each of the four different types of calli (0.5 g) was placed in a pre-cooled mortar. During this period, liquid nitrogen was continuously added while grinding the calli thoroughly. The ground samples were transferred to a 10 ml centrifuge tube, and 8 ml of precooled 50 mmol/L phosphate buffer with pH 7.8 (containing 1% PVP-40 and 0.1 mmol/L EDTA) was added. Samples were centrifuged (9500 × *g* 15 min) and the supernatant (10 ml) taken as the crude enzyme solution and stored at 4 °C until further use. Coomassie blue [13], NBT photoreduction [14], guaiacol [15], and UV spectrophotometry [16,17], were used to determine protein content, SOD activity (Superoxide Dismutase), POD activity (Peroxidase), CAT activity (Catalase from Micrococcus Lysodeikticus), and multi-PPO activity (Polyphenol Oxidase) of crude enzyme solutions from different types of *E. urophylla × E. grandis* calli.

### Plant hormone extraction and determination

Different types of calli of *E. urophylla × E. grandis* (2 g) were frozen in liquid nitrogen, Trizol reagent was added, and mixed evenly. Three biological replicates of each group of calli samples were stored on dry ice and sent for the determination of endogenous hormones (De novo, Guangzhou, China). The pretreatment steps for different types of calli are described by Cai, Niu, and Xiao et al. [18–20]. The data acquisition system comprised ultra-high performance liquid chromatography and tandem mass spectrometry (MS/MS). The liquid-phase conditions were based on the methods of Pan, Šimura, and Cui et al. [21–23].

### Analysis of the efficiency of RT-qPCR amplification of GRFs expression

The total RNA obtained from fresh calli of four different types of *E. urophylla × E. grandis* (200 mg) was isolated using TRIzol reagent (Thermo Fisher Scientific, USA), with the quality and quantity of the isolated RNA being determined using a NanoDrop spectrophotometer (Thermo Fisher Scientific) and agarose gel electrophoresis, respectively.The RNA concentration and the ratio of OD_260_/OD_280_ being determined, which reached the standard for qRT-PCR experiments.

First-strand cDNA was synthesized from the isolated RNA using a Maxima First Strand cDNA Synthesis Kit (Thermo Fisher Scientific), and double-stranded cDNA was subsequently synthesized.The efficiency of real-time quantitative polymerase chain reaction (RT-qPCR) amplification of growth-regulating factor (*GRF*) genes was analyzed following the TransStart Tip Green qPCR SuperMix instructions of TransGen Biotech. Gene names and sequences of the RT-qPCR primers used in this study are listed in Table 1.

**Table 1:** Primer sequences for *GRF* family genes.

Primer name	Forward sequence (5’-3’)	Reverse sequence (5’-3’)
*Actin*	TTGTGCTCAGTGGTGGAACC	TCTGCCTTTGCGATCCACAT
*EuGRF1*	TAACCACCCAAACCATCCTC	AACCTCGCCCACATCAGCAA
*EuGRF3*	GGTCGCTCCAGGATTCTGACAACT	GCGTCCCTGTCGTTCGTGCT
*EuGRF5*	GCACCTGCCGACCTCTTATC	TTGTTTCTGCTGCTTTGACG
*EuGRF6*	CCTCCGAGCCTGTCTATGTT	AGTTTCGGGAGTCTAACAAGGA
*EuGRF7*	GGGATTCATTGATGCCTGGTC	ATTATTGCCACCCACGGACA
*EuGRF8*	GGCTCTGCCTCTGCTGATTC	CTTGCCACTGTGCTGCTGTAA

### Total RNA extraction, cDNA library preparation and transcriptome sequencing

Total RNA was extracted using Trizol reagent kit (Invitrogen, Carlsbad, CA, USA) according to the manufacturer’s protocol. RNA quality was assessed on an Agilent 2100 Bioanalyzer (Agilent Technologies, Palo Alto, CA, USA) and checked using RNase free agarose gel electrophoresis. After total RNA was extracted, eukaryotic mRNA was enriched by Oligo (dT) beads, while prokaryotic mRNA was enriched by removing rRNA by Ribo-ZeroTM Magnetic Kit (Epicentre, Madison, WI, USA). Then the enriched mRNA was fragmented into short fragments using fragmentation buffer and reverse transcripted into cDNA with random primers. Second-strand cDNA were synthesized by DNA polymerase I, RNase H, dNTP and buffer. Then the cDNA fragments were purified with QiaQuick PCR extraction kit (Qiagen, Venlo, The Netherlands), end repaired, poly(A) added, and ligated to Illumina sequencing adapters. The ligation products were size selected by agarose gel electrophoresis, PCR amplified, and sequenced using Illumina HiSeq400 by Gene Denovo Biotechnology Co. (Guangzhou, China) [5,24].

### Bioinformatics analysis of the RNA-seq data

In order to ensure the data quality, the original data should be filtered before information analysis to reduce the analysis interference caused by invalid data. First, fastp [25] is used to control the quality of the original data, remove low-quality data (data containing adapter, data containing more than 10% N, and data containing all A bases), and obtain effective data. The clean reads were aligned to the *Eucalyptus* genome version GCA_000612305.1 using the HISAT2.2.4 software. According to the comparison results of HISAT2.2.4, the transcript was reconstructed by string tie [11], and the expression of all genes in each sample was calculated using RESM [26]. The input data for differential gene expression analysis were the read count data obtained from gene expression level analysis, which was analyzed using DESeq2 software [27]. Based on the results of the difference analysis, we screened the genes with a false discovery rate (FDR) <0.05, and | log2 fold change (log_2_FC)| > 1 as significantly different genes. Bioinformatic analysis such as correlation analysis and PCA analysis were conducted using Omicsmart, a real-time interactive online platform for data analysis (http://www.omicsmart.com). Then we conducted the Kyoto Encyclopedia of Genes and Genomes (KEGG) enrichment analysis and Gene Ontology (GO) enrichment based on the DEGs of each pairwise comparison (False discovery rate < 0.05). Protein-Protein interaction network was identified using String v10 [28], which determined genes as nodes and interaction as lines in a network. The network file was visualized using Cytoscape (v3.7.1) software [29] to present a core and hub gene biological interact.

## Results

### Callus induction results

**Fig 1:**
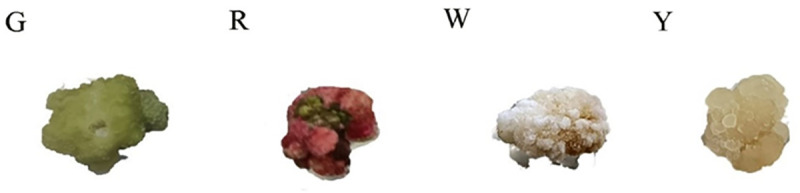
Four different types of calli of *E. urophylla* × ***E. grandis*** **induced under different conditions.**

Green, red, white, and yellow calli of *E. urophylla × E. grandis* obtained under different conditions are shown in Fig 1. It can be seen from the figure that the calli of *E.urophylla × E.grandis* differentiated into four different colors - green, red, white and yellow. The texture of the green callus was hard, small particles were differentiated on the surface, and the whole callus was dense and moist. Although the red callus was mixed with the green granular callus in the middle, it was red as a whole, the peripheral water content was low, and the middle water content was high. The white callus appeared to be wrapped by a layer of fine white powder, and the whole was loose and dry. The yellow callus was crystal clear, and the water content was the highest among the four types of calli.

### The results of enzymes assays

**Fig 2:**
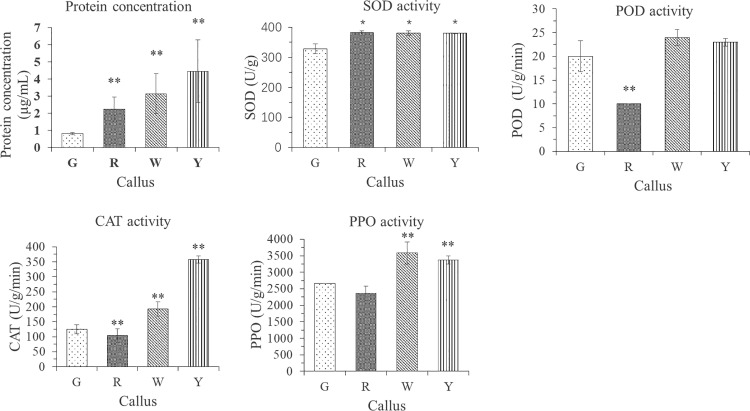
Differences in physiological indices in the four different types of calli.

Fig 2 shows five evaluation physiological indexes of green, red, white and yellow *E. urophylla × E. grandis* calli obtained under different conditions. The protein content of each calli increased in turn, and the increasing range was relatively stable. Compared with the protein content of green *E. urophylla × E. grandis* calli, the protein content of red, white, and yellow calli reached a significantly higher level (P < 0.01).

The SOD activities of *E. urophylla × E. grandis* were calculated from the measured OD_560_. The SOD activities of red, white, and yellow calli were similar but all were higher than that of green calli, the differences from the green were significant (0.01 < P < 0.05).

The POD activities of calli of different colors of *E. urophylla × E. grandis* were calculated from the measured OD_470_. The POD activity of red callus of *E.urophylla × E.grandis* was significantly lower than that of green callus, and the difference reached a very significant level (P < 0.01); The POD activity of white and yellow callus was higher than that of green callus, and the difference was not significant (P > 0.05).

The CAT activity of calli of different colors of *E. urophylla × E. grandis* was calculated from the measured OD_240_. The CAT activity of the red calli of *E. urophylla × E. grandis* was significantly lower than that of the green calli (P < 0.01), and the POD activity of the white and yellow calli was significantly higher than that of the green calli (P < 0.01).

According to, the PPO activity of calli of different colors of *E. urophylla × E. grandis* was calculated from the measured OD_420_. The PPO activity of red calli was lower than that of green calli, and the difference was not significant (P > 0.05). The PPO activity of white and yellow calli was significantly higher than that of green calli (P < 0.01).

### Differences in plant hormones in the four different types of calli

**Fig 3:**
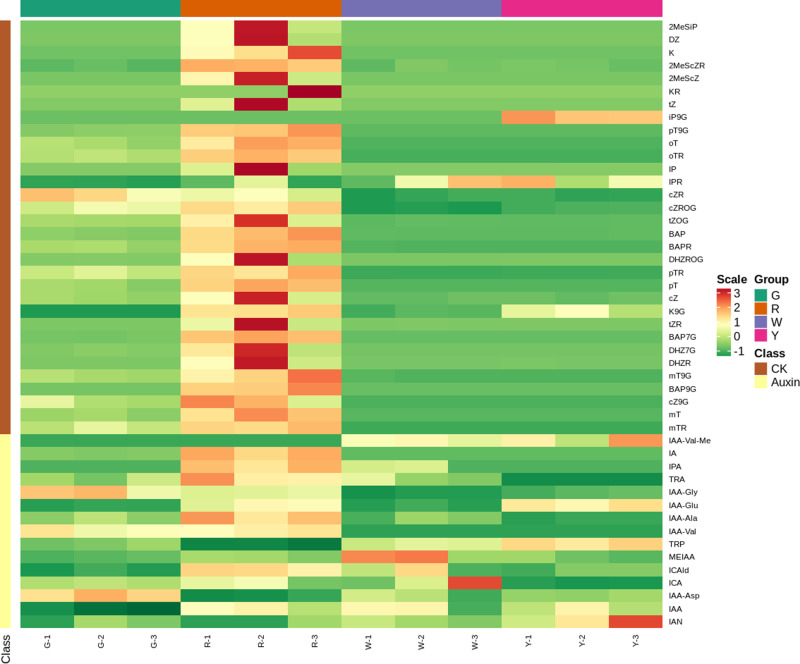
Overall clustering diagram of samples.

The obtained data were standardized, the clustering heat map of the four samples was analyzed, and an R program script was used to draw the clustering heat map, as shown in Fig 3. In general, the expression of the same cluster in the red calli was much higher than that in the other three calli. Locally, the clusters of indoleacetic acid, aspartic acid, isopentene adenine nucleoside, indole-3-acetonitrile, isopentene adenine-9-glucoside, indoleacetic acid valine methyl ester, and L-tryptophan in the red and green calli were significantly different from those in the white and yellow calli. Cis-zeatin D-riboside, indoleacetic acid glycine, cis-zeatin-O-glycoside, N-(4-methoxybenzyl)-adenosine, N-(3-hydroxybenzyl) adenosine and other clusters were obviously different from the other two calli in green and red calli. The clustering of indoleacetic acid valine and indole-3-carboxylic acid in green and red calli was significantly different from that in the yellow and white calli.

### (RT)-qPCR amplification result of GRFs expression

**Fig 4:**
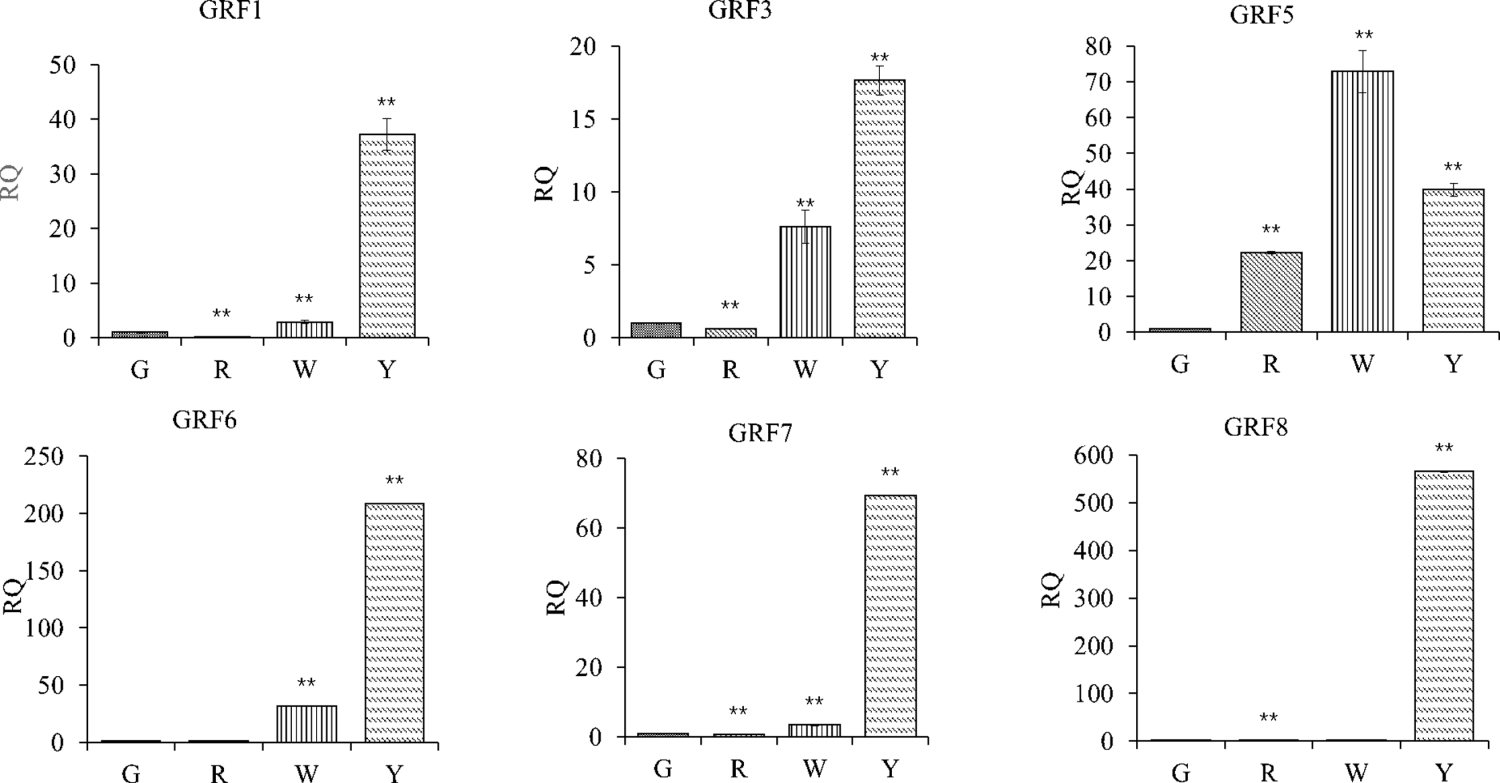
Differences of GRFs gene expression in different types of *E. urophylla ×  E. grandis callus.*

Fig 4 shows the changes in the expression level of GRFs gene family verified by real-time fluorescence quantitative PCR.

Different *GRFs* play different regulatory roles in the morphological differences of *E. urophylla × E. grandis* callus, and the expression of *GRFs* is closely related to the regulation of plant hormones, which means that the hormone levels in different callus formations and differentiations are uneven.

### Transcriptome sequencing and expression analysis of four different types of callus

A total of 51836582, 44657346, 55047483, and 49740461 clean reads were generated for the green, red, white, and yellow *E. urophylla × E. grandis* callus samples, respectively. Alignment of clean reads to the *E. grandis* reference genome yielded mapping rates of 88.02%, 87.59%, 88.37%, and 88.22%, respectively, indicating that in these four cases, a high proportion of reads were mapped to the reference gene (*Eucalyptus* genome). A total of 86.38 Gb of bases were obtained from the test samples. After filtration, the base number of each sample was more than 6.0 Gb, the percentage of clean data was more than 99.4%, the GC content of each sample was more than 49%, the quality of Q20 was more than 97%, and the quality value of Q30 was more than 92%, which met the quality requirements of subsequent analysis (Table 2).

**Table 2:** Statistical table of base information.

Sample	Raw data (Gb)	Clean data (Gb)	AF_Q20 (%)	AF_Q30 (%)	AF_N (%)	AF_GC (%)
G-1	7.71	7.64	97.56	93.37	0.00	49.20
G-2	6.99	6.92	97.44	93.04	0.00	49.25
G-3	7.55	7.48	97.59	93.39	0.00	49.30
R-1	6.77	6.71	97.60	93.49	0.00	49.22
R-2	6.10	6.04	97.60	93.43	0.00	49.39
R-3	6.29	6.23	97.62	93.53	0.00	49.30
W-1	8.18	8.10	97.59	93.39	0.00	49.30
W-2	8.09	8.01	97.64	93.49	0.00	49.19
W-3	7.35	7.28	97.64	93.46	0.00	49.13
Y-1	7.20	7.13	97.64	93.53	0.00	49.10
Y-2	7.15	7.08	97.62	93.51	0.00	49.29
Y-3	6.99	6.91	97.62	93.52	0.00	49.31

G. Green callus, R. red callus, W. white callus, Y. yellow callus.

**Fig 5:**
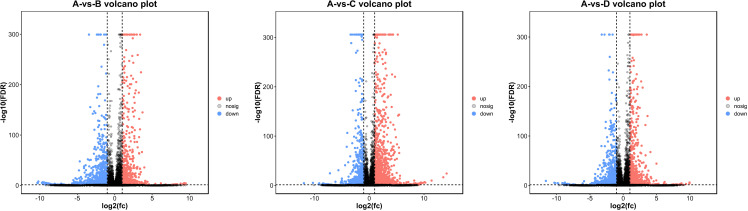
Difference comparison volcano map.

Through differentially expressed gene (DEG) screening (FC ≥ 2, FDR < 0.05), it can be seen that compared with the callus of green *E. urophylla × E. grandis*, there were 2203 DEGs in the callus of red *E. urophylla × E. grandis*, including 764 upregulated genes and 1439 downregulated genes; 2485 DEGs were identified in the callus of *E. urophylla × E. grandis*, including 1493 upregulated genes and 992 downregulated genes, and 2078 DEGs in the callus of *E. urophylla × E. grandis*, of which 977 were upregulated and 1101 were downregulated (Fig 5).

### Functional analysis and enrichment of DEGs

**Fig 6:**
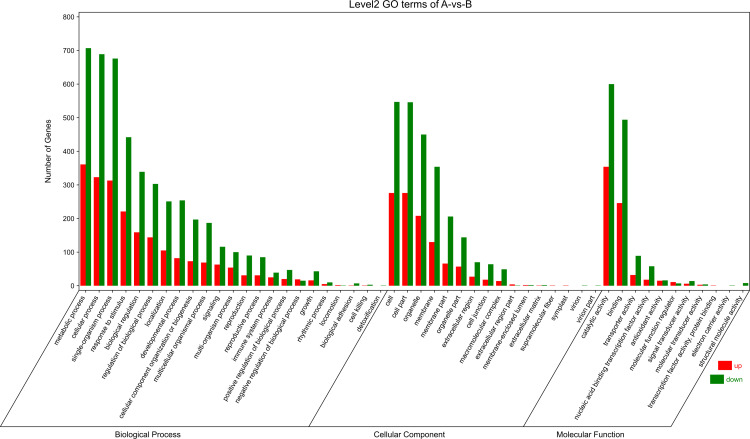
Histogram of GO enrichment and classification of green and red callus.

**Fig 7:**
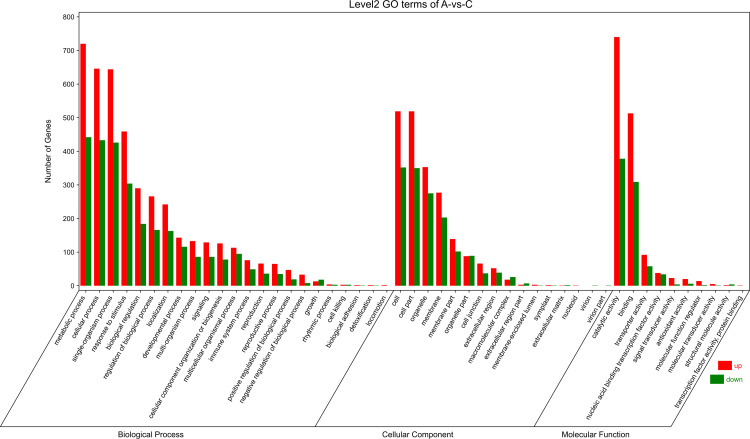
Histogram of GO enrichment and classification of green and white callus.

**Fig 8:**
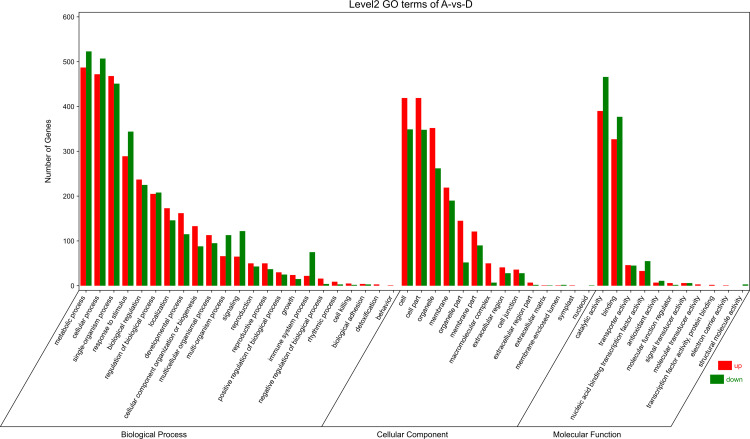
Histogram of GO enrichment and classification of green and yellow callus.

To explore the correlation of DEGs in different types of calli of *E. urophylla × E. grandis*, we performed GO annotation of the expressed genes, the results of which are shown in Figs 6–8.

The genes predicted to be enriched in the cellular component of green vs red callus were “GO:0005576~extracellular region”, “GO:0030312~external encapsulating structure” and “GO:0071944~cell periphery”. Those Involved in specific molecular functions were “GO:0046906~tetrapyrrole binding”, “GO:0016491~oxidoreductase activity” and “GO:0043169~cation binding”. Meanwhile, those involved in biological processes were “GO:0010410~hemicellulose metabolic process”, “GO:0045491~xylan metabolic process” and “GO:0009832~plant-type cell wall biogenesis”. The top upregulated and downregulated DEGs in green and red calli were mainly associated with metabolic processes, cellular processes, single-organism processes, responses to stimuli, catalytic activity, binding, cell, cell part, and organelle.

In green vs white callus, the genes predicted to be enriched in the cellular component were “GO:0071944~cell periphery”, “GO:0030312~external encapsulating structure” and “GO:0005576~extracellular region”. Those involved in specific molecular functions were “GO:0016491~oxidoreductase activity”, “GO:0016705~oxidoreductase activity, acting on paired donors, with incorporation or reduction of molecular oxygen” and “GO:0046906~tetrapyrrole binding”. While those involved in biological processes were “GO:0001101~response to acid chemical”, “GO:0010243~response to organo-nitrogen compound” and “GO:0006820~ anion transport”. The top upregulated and downregulated DEGs in green and white calli were found to be mainly associated with metabolic processes, cellular processes, single-organism processes, responses to stimuli, biological regulation, catalytic activity, binding, cell, cell part, organelle, membrane, etc.

In green vs yellow callus, the genes predicted to be enriched in the cellular component were “GO:0044436~thylakoid part”, “GO:0009579~thylakoid” and “GO:0044434~chloroplast part. Those with specific molecular functions were “GO:0016491~oxidoreductase activity”, “GO:0016705~oxidoreductase activity, acting on paired donors, with incorporation or reduction of molecular oxygen” and “GO:0046906~tetrapyrrole binding”. While those involved in biological processes were “GO:0010243~response to organo-nitrogen compound”,“GO:0009719~response to endogenous stimulus” and “GO:0002252~immune effector process”. The top upregulated and downregulated DEGs in green and yellow calli were mainly associated with metabolic processes, cellular processes, single-organism processes, responses to stimuli, biological regulation, catalytic activity, binding, cell, cell part, organelle, and membrane.

Furthermore, we compared the DEGs with diverse regulations in the four different types of calli of *E. urophylla × E. grandis*. Compared with green calli, ATL71, CAD, EARLI1, GP1, IPT1, PER3, MYB4, At2g39510, CAS1, HSP70, and other gene families were significantly upregulated; GLP6, IRX12, XCP1, WDL3, DIR23, DTX42, CYP71A9, DOGL4, CYP94A2, XI-F, HIPP39, PUP21, LOG1, BZIP43, At3g05950, and other gene families were significantly downregulated in yellow calli. White and yellow calli were analyzed identically. AtMg01250, EXLB1, VIT_14s0108g01050, At2g39510, HHT1, GLO4, CAD, EARLI1, MYB2, and other gene families were significantly upregulated, whereas NRAMP5, At3g05950, MonoTPS1, HIPP39, ML6, PYRC5, At5g33370, rnf144a, CYP75B2, and other gene families were significantly downregulated in the white calli. AOP1, EARLI1, SAUR23, At1g32780, AOP1.2, DIR21, ndhU, RL6, TOM1, GP1, and other gene families were significantly upregulated, whereas AGP31,GLP6, ERF2, ERF13, ERF020, At4g22030, MYB41, CXE12, At5g39110, PLP2, CAND7, and other gene families were significantly downregulated in the yellow calli.

**Fig 9:**
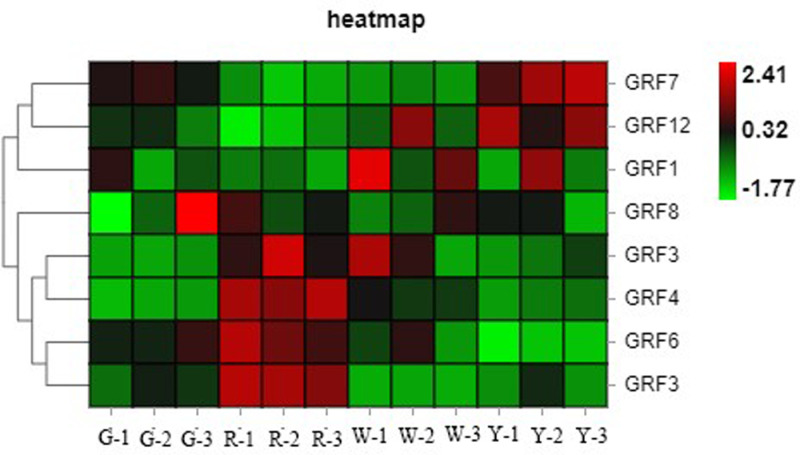
Differential expression heat map of GRFs in different types of callus.

The expected gene growth regulators, GRFs, were screened from the differentially expressed genes and their heat maps were drawn (Fig 9). It was found that the clustering of GRFs genes in different types of *E. urophylla × E. grandis* and calli was significantly different. The expression levels of GRF3, GRF4, and GRF6 in the red calli were much higher than those in the other three calli, while the expression levels of GRF7 and GRF12 in the yellow calli were also much higher than those in the other three calli. miRNA396 is highly conserved in plants, and mainly participates in biological activities by regulating the expression of target genes. It is also involved in plant stress resistance. GRFs are the main target gene of miRNA396. Based on the differential expression of GRFs in the heat map, it can be preliminarily concluded that the life activities of different calli are different, and the difference in morphology is closely related to the difference in gene expression.

Based on the above GO enrichment analysis of differentially expressed genes, we found that the DEGs were mostly concentrated in the same biological process. These data indicate that the different calli morphologies are mainly associated with a high level of metabolic activity, cellular processes, genetic information processes, organismal systems, and environmental information processes.

To better understand the functional role of the DEGs in the regeneration process of *E. urophylla × E. grandis*, KEGG pathway analysis of the specific common DEGs of the four different types of *E. urophylla × E. grandis* calli was conducted. Compared with green calli, 7021 DEGs with functional annotations were involved in 439, 503, and 364 metabolic pathways in red, white, and yellow calli, respectively. Metabolic pathways, biosynthesis of secondary metabolites, phenylpropanoid biosynthesis, plant hormone signal transduction, carbon metabolism, plant-pathogen interaction, and MAPK signaling pathway were seven of the most significantly enriched pathways in the four different types of *E. urophylla × E. grandis* calli. Moreover, the metabolic pathway was the most significantly enriched pathway for the DEGs shared in the four different types of calli, suggesting that this pathway positively influences the regeneration of *E. urophylla × E. grandis*. The top five significantly enriched pathways for the specific common DEGs of the four different types of *E. urophylla × E. grandis* calli were listed in Table 3.

**Table 3:** Pathway enrichment.

Type	Pathway	Number of candidate genes	P-value	Q-value	Pathway ID
A Vs B	Metabolic pathways	280	0	0	ko01100
A Vs B	Biosynthesis of secondary metabolites	167	0.000003	0.00008	ko01110
A Vs B	Phenylpropanoid biosynthesis	53	0	0.00001	ko00940
A Vs B	Plant hormone signal transduction	37	0.001812	0.032919	ko04075
A Vs B	Carbon metabolism	32	0.065082	0.332719	ko01200
A Vs C	Metabolic pathways	305	0	0	ko01100
A Vs C	Biosynthesis of secondary metabolites	209	0	0	ko01110
A Vs C	Phenylpropanoid biosynthesis	53	0.000017	0.000361	ko00940
A Vs C	Plant hormone signal transduction	39	0.005938	0.048416	ko04075
A Vs C	Plant-pathogen interaction	39	0.042409	0.17309	ko04626
A Vs D	Metabolic pathways	222	0	0	ko01100
A Vs D	Biosynthesis of secondary metabolites	140	0.000011	0.000182	ko01110
A Vs D	Phenylpropanoid biosynthesis	47	0	0.000005	ko00940
A Vs D	Plant hormone signal transduction	31	0.003609	0.033468	ko04075
A Vs D	MAPK signaling pathway - plant	27	0.000649	0.008278	ko04016

A. green callus, B. red callus, C. white callus, D. yellow callus.

The genes found in this study might be associated with callus development and differentiation in *Eucalyptus*. Further experiments are required to explore these functions.

## Discussion

Callus induction is considered to be a key precursor to adventitious bud formation, in this study, we analyzed the transcriptome profiles of four different types calli from *E. urophylla × E. grandis*. Transcriptome analysis revealed that there were significant differences in the clustering of differentially expressed genes. Compared with the green calli, 2203, 2485, and 2078 differentially expressed genes were identified in the red, white, and yellow calli, respectively. Through differentially expressed gene (DEG) screening (FC ≥ 2, FDR < 0.05), it can be seen that compared with the callus of green *E. urophylla × E. grandis*, there were 2203 DEGs in the callus of red *E. urophylla × E. grandis*, including 764 upregulated genes and 1439 downregulated genes; 2485 DEGs were identified in the callus of *E. urophylla × E. grandis*, including 1493 upregulated genes and 992 downregulated genes, and 2078 DEGs in the callus of *E. urophylla × E. grandis*, of which 977 were upregulated and 1101 were downregulated.The genes identified in this study may play vital roles in callus development and may be related to the vegetative reproductive ability of *Eucalyptus*.

Although plant organ culture technology has primacy over traditional vegetative propagation methods because it has a high proliferation rate, some problems remain, such as explants obtained from plants in the external environment that are easily polluted, callus browning, and vitrification [30]. Many miRNAs may participate in *Eucalyptus* somatic embryogenesis and affect embryonic callus development [6–10]. We analyzed the expression level of GRFs gene family which is regulated by miR396 [31]. It was found that the clustering of GRFs genes in different types of *E. urophylla × E. grandis* and calli was significantly different. Based on the differential expression of GRFs in the heat map, it can be preliminarily concluded that the life activities of different calli are different, and the difference in morphology is closely related to the difference in gene expression. miRNA396 is highly conserved in plants, and mainly participates in biological activities by regulating the expression of target genes.Differences in *Eucalyptus* genotypes, explant materials, and the proportion of hormone species are important factors affecting the regeneration of *Eucalyptus* tissue culture [32]. Plant growth regulators play important roles in callus morphological construction. Callus can produce a large amount of ethylene in the process of culture and is regulated by growth regulators in the medium. Some physiological effects of auxin and cytokinin on calli may be mediated by ethylene [33].

Compared with the callus of *Olea europaea* under light treatment, the activities of antioxidant enzymes (SOD, POD, CAT, and APX) in the callus grown under dark conditions were significantly higher [34]. At present, studies have shown that H_2_O_2_, as an intracellular signal transduction substance, may affect the regulatory expression of genes through the cellular signal transduction system and has the potential to regulate gene expression and promote embryonic development [35]. The expression of genes related to morphology and structure in a specific temporal and spatial order is the essence of callus differentiation into different structures and morphologies, and the expression of some genes is affected by free-radical metabolism. In organisms, the protective system composed of pods, SOD, CAT, and other enzymes regulates the balance of oxygen free radicals in cells [36].

The dysregulation of these genes in this study may indicate that they may play a crucial role in the beginning of wounding and callus differentiation to promote healing and prepare for rapid downstream development. According to our transcriptome results, genes related to callus morphology, such as hormone regulatory, photosynthesis-related, and antioxidant enzyme isoenzyme genes, can be further studied and verified. Gene imbalance is regulated by several mechanisms and the interaction network requires further exploration.

## Conclusions

Induction of calli from explants is the first step in somatic embryogenesis. Our preliminary study provides insights into the molecular mechanisms of *E. urophylla × E. grandis* explants that generate embryogenic calli and regenerate into intact plants. The findings of this study provide a basis for the following directional and efficient genetic improvement of *Eucalyptus* using genetic engineering technology and provide a direction for optimizing *Eucalyptus* material and resistance and shortening the breeding cycle of new *Eucalyptus* varieties.

## Abbreviations

*E. urophylla × E. grandis*: *Eucalyptus urophylla* × *Eucalyptus grandis*
DEGs: differentially expressed genes
GRF: growth regulating factor
GO: gene ontology
KEGG: Kyoto Encyclopedia of Genes and Genomes
FC: fold change
FDR: false discovery rate
SOD: superoxide dismutase
POD: peroxidase
CAT: catalase from micrococcus lysodeikticus
PPO: polyphenol oxidase
PCR: polymerase chain reaction
IAA: indole-3-acetic acid
PBU: N-phenyl-N’-[6-(2-chlorobenzothiazol)-yl] urea
OD: optical density.
